# Early Haploidentical Hematopoietic Stem Cell Transplantation Provides Rapid Leukocyte and Immune Reconstitution in AK2 Patient Identified by TREC Newborn Screening

**DOI:** 10.1007/s10875-025-01863-5

**Published:** 2025-02-11

**Authors:** Alphan Cicek, Friedhelm R. Schuster, Janel O. Boyle, Manfred Hoenig, Roland Meisel, Sujal Ghosh

**Affiliations:** 1 Division of Pediatric Stem Cell Therapy, Department of Pediatric Oncology, Hematology and Clinical Immunology, Medical Faculty, Center of Child and Adolescent Health, Heinrich-Heine-University, Moorenstraße 5, 40225 Düsseldorf, Germany; 2 Department of Pediatrics, Division of Allergy, Immunology, and Bone Marrow Transplantation, University of California San Francisco, San Francisco, CA USA; 3 Department of Clinical Pharmacy, University of California San Francisco, San Francisco, CA USA; 4 Department of Pediatrics, University Medical Center Ulm, Ulm, Germany

## Abstract

Reticular dysgenesis (RD) is a rare inborn error of immune cell formation defined by severe combined immunodeficiency, agranulocytosis and sensorineural deafness. We report a case of successful haploidentical maternal hematopoietic stem cell transplantation (HSCT) in a boy with RD detected by TREC newborn screening. The patient was admitted to our hospital at 2 weeks of age and was kept in laminar-air flow / hepa-filtered isolation until HSCT was performed at 8 weeks of age with a busulfan, fludarabine conditioning regime. Except few episodes of acute skin graft-versus-host disease (aGVHD) the peritransplant period was uneventful. The patient was discharged 7 weeks post-HSCT. At 18 months of age cochlear implants were placed. The patient was thriving well, showed full donor chimerism and a T cell count > 1000 TCRab + CD3 + cells/µl after one year. Our case highlights that severely immune-compromised patients with RD benefit from early diagnosis by newborn screening, immediate isolation to prevent infections, and early haploidentical HSCT to overcome neonatal neutropenia and establish protective immunity.

## To the Editor

Reticular dysgenesis (RD) is a rare inborn error of immunity defined by the clinical triad of severe combined immunodeficiency (SCID), agranulocytosis, and sensorineural deafness. The disease is caused by mutations in (adenylate kinase) *AK2*, which has a central role in cellular energy and adenine nucleotide metabolism [1–3]. While the exact mechanism remains to be elucidated, most groups agree that differentiation and proliferation of haematopoietic cells is severely compromised at an early stage. The dysregulation of potassium and adenosine 5’- triphosphate concentrations in the inner ear endolymph is hypothesized to result in sensorineural deafness [2].

**Fig. 1:**
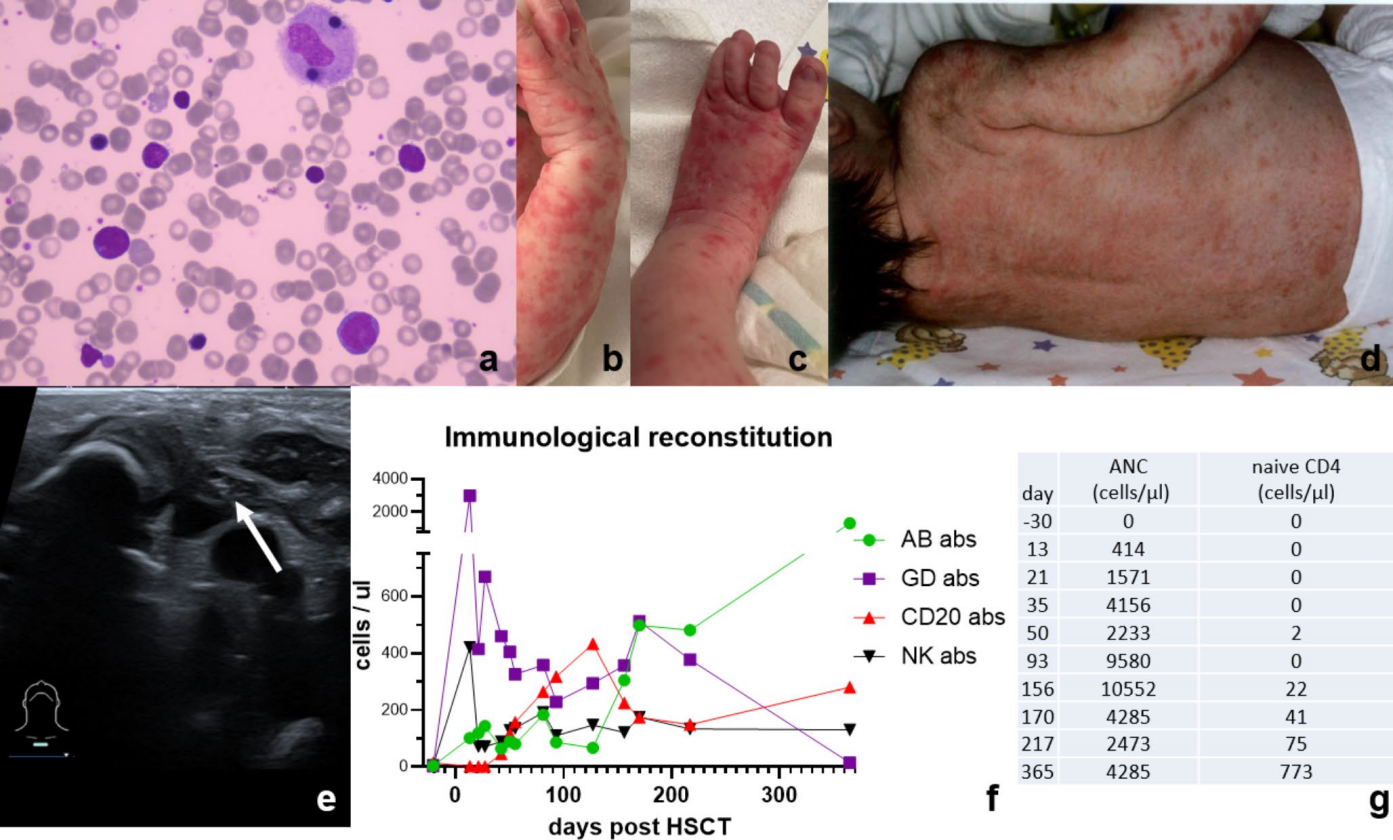
**a**. Bone marrow aspirate showing normal red blood cells and platelets. Few maturing lymphocytes are seen, however myeloid maturation is not beyond through the blast / promyelocyte stage, neutrophil granulocytes are absent. **b**-**d**. Skin manifestations after birth: maculopapular rash on trunk and distal upper and lower limbs as signs of maternal GvHD or bacterial superinfections **e**. Thymic ultrasound at admission indicating a small thymus (white arrow) remnant suggestive of severe combined immunodeficiency **f**. Immunological reconstitution after HSCT: The chart shows the course of the immunological reconstitution in our transplanted reticular dysgenesis patient. Left y-axis: CD3 gamma/delta (■), CD3 alpha/beta (●), CD20 (▲), and NK (▼) cell count. As known in TCRab/CD19 depleted haplo SCT the immediate CD3 increase reflects expansion of TCRgd T cells. The increase is notably higher than in other TCRab/CD19 haplo SCT performed in screened SCID patients. All cells are consistently of donor origin. **g**. ANC and naive CD4 cell count before and after transplantation

Hematopoietic stem cell transplantation (HSCT) is the only option to cure this otherwise fatal disease. Transplant outcome has been insufficient with 6 of 17 deceased patients in a T cell depleted setting in an international survey. Nine required a secondary procedure [4]. We report a case of successful haploidentical hematopoietic stem cell transplantation in a boy of Turkish consanguineous descent suffering from RD. The infant (born at 37 weeks of gestation after an uneventful pregnancy) was admitted at our centre 10 days after birth following confirmed abnormal TREC newborn screening (TREC 0). Notably, newborn hearing screening also suggested further evaluation of hearing. Initial white blood count showed aleukocytosis with absent neutrophils and lymphocytes. The patient was immediately put into a hepa-filtered and laminar-air flow equipped room. Breastfeeding was stopped. Panel-based whole exome sequencing confirmed previously reported homozygotic mutations (453delC/ Tyr152ThrfsX12) in *AK2* [3]. A bone marrow aspirate showed a maturation arrest at the promyelocyte stage, while the erythroid and megakaryocytic lineages were not affected (Fig. 1a). Hemoglobin and platelet count were normal. Cytogenetic evaluation of the bone marrow did not reveal any abnormalities.

During the further course a fine-spotted maculopapular rash appeared on the trunk and distal upper and lower limbs, initially suggesting the possibility of bacterial skin infections. However, maternal graft-versus-host-disease (GvHD) was considered after maternal T cells were detected in the peripheral blood via XY fluorescense-in-situ-hybridisation 13 days after birth (Fig. 1b-d). Apart from dermatological affections he was in a good general condition. Ultrasound of the thymus revealed a hypoplastic organ (Fig. 1e). Based on high clinical urgency with missing white blood cells and lack of a potential sibling donor we decided to bypass search for an HLA-matched unrelated donor and opt for immediate haploidentical stem cell transplantation.

HSCT was performed at age of eight weeks with TCRab/CD19-depleted maternal peripheral blood stem cells (32,9 × 10^6^ CD34 cells/kg, 2,1 × 10^4^ TCRab + CD3 + cells/kg). A myeloablative conditioning consisting of busulfan (TDM AUC 75 mg*h/l) and fludarabine (predicted AUC 16 mg*h/l) on d-8 to d-5 was applied, GvHD prophylaxis consisted of Anti-thymocyte globulin (Grafalon^®^) 30 mg/kg (d-11 to d-9) and mycophenolate mofetil (d-1 to d + 30). Platelet engraftment (> 50.000 platelets/µl) was achieved on day + 13, neutrophil engraftment on day + 15. Chimerism analysis of the peripheral blood showed full donor chimerism on day + 20. From day + 11 onwards our patient suffered from recurrent episodes of isolated acute graft versus host disease of the skin (max. stage 3 rash, grade II) which required systemic and topical steroids. Seven weeks post SCT we were able to discharge the patient, however repeated admission were required due to skin GvHD flares treated with intermittent systemic steroids.

At six months of age, we could discontinue all anti-infective prophylaxis (acyclovir, cotrimoxazole, voriconazole) and home-isolation. The patient is now steroid-free for one year (18 months post-SCT) and GvHD has not re-emerged. The patient thrives well along his percentiles. Early childhood development was age-appropriate, however due to bilateral sensorineural deafness language development has been delayed. Treatment with cochlear implants was performed at the age of 18 months. Latest immunology results showed full donor chimerism and a T cell count with > 1000 TCRab + CD3 + cells/µl and a naïve CD4 cell number > 500/µl (for full reconstitution data see Fig. 1f g). The patient is without prophylaxes and without immunoglobulin replacement therapy (last IgG substitution on day + 155). Furthermore B cells have started to differentiate one year after HSCT (10% of B cells express CD27).

## Conclusion

We report on the outcome of the first RD patient identified in the German newborn screening since its implementation five years ago. Individual RD cases in other screening programs have not been reported in depth. It may be debatable whether infants with RD are diagnosed early enough to prevent their first life-threatening infection in the early postnatal period, and thus benefit from newborn screening [2, 4, 5]. Our case impressively illustrates that immediate isolation may protect from infections. Immediate haploidentical TCRab/CD19 depleted HSCT confers protective immunity to overcome neonatal neutropenia and lymphopenia quickly in a disease which has proven poor outcome in symptomatic children even if they receive alloSCT [4]. Key features contributing to the success of this treatment include a high CD34 + cell count and a fully myeloablative conditioning. Low residual TCRab cells and an Anti-thymocyte globulin / mycophenolate mofetil prophylaxis strategy reduced the likelihood of GvHD. Despite CMV positivity of the mother the patient never experienced any CMV or other viral (re)activation. However it should be noted that there are emerging reports, which observe severe viral infections even in screened SCID infants despite laborious efforts to avoid infections. This report represents a single case, and the potential success of this approach requires confirmation through future research involving additional cases.

## Data Availability

No datasets were generated or analysed during the current study.
